# Biological indicators of chemoresistance: an ex vivo analysis of γH2AX and *p53* expression in feline injection-site sarcomas

**DOI:** 10.1186/s12935-018-0690-0

**Published:** 2018-11-22

**Authors:** Yike Bing, Zacharie Wund, Tina Abratte, Lucia Borlle, Susie Kang, Teresa Southard, Kelly R. Hume

**Affiliations:** 1 Department of Clinical Sciences, College of Veterinary Medicine, Cornell University, Ithaca, NY USA; 2 Department of Biomedical Sciences, College of Veterinary Medicine, Cornell University, Ithaca, NY USA

**Keywords:** Chemoresistance, Sarcoma, Animal model, GammaH2AX, DNA damage

## Abstract

**Background:**

The response of soft tissue sarcomas to cytotoxic chemotherapy is inconsistent. Biomarkers of chemoresistance or chemosensitivity are needed in order to identify appropriate patients for treatment. Given that many chemotherapeutics kill cells through direct DNA interactions, we hypothesized that upregulation of DNA damage response mechanisms would confer resistance to cytotoxic chemotherapy in sarcomas. To study this, we used spontaneously-occurring feline injection-site sarcomas (FISS).

**Methods:**

γH2AX and *p53* expression were determined in biopsy samples of FISS. γH2AX expression was determined via immunohistochemistry whereas *p53* expression was determined via qRT-PCR. Cell lines derived from these sarcoma biopsies were then treated with carboplatin (*N *= 11) or doxorubicin (*N *= 5) and allowed to grow as colonies. Colony forming-ability of cells exposed to chemotherapy was compared to matched, untreated cells and expressed as percent survival relative to controls. ImageJ was used for quantification. A mixed model analysis was performed to determine if an association existed between relative survival of the treated cells and γH2AX or *p53* expression in the original tumors. Cell lines were validated via vimentin expression or growth as subcutaneous sarcomas in nude mice.

**Results:**

An association was detected between γH2AX expression and relative survival in cells exposed to carboplatin (*P *= 0.0250). In the 11 FISS tumors evaluated, γH2AX expression ranged from 2.2 to 18.8% (mean, 13.3%). Cells from tumors with γH2AX expression higher than the sample population mean had fourfold greater relative survival after carboplatin exposure than cells from tumors with γH2AX expression less than the mean. There was no association between relative survival after carboplatin exposure and *p53* expression (*P *= 0.1608), and there was no association between relative survival after doxorubicin exposure and either γH2AX (*P *= 0.6124) or *p53* (*P *= 0.8645) expression. Four cell lines were validated via growth as sarcomas in nude mice. Vimentin expression was confirmed in the other 7 cell lines.

**Conclusions:**

γH2AX expression, but not wild type *p53*, may potentially serve as a biomarker of resistance to platinum therapeutics in soft tissue sarcomas. To further investigate this finding, prospective, in vivo studies are indicated in animal models.

## Background

Soft tissue sarcomas (STS) are an anatomically and histologically diverse group of solid malignancies of mesenchymal origin. These tumors are relatively rare, with an estimated 12,000 new cases and approximately 5000 deaths in the United States each year [1]. Surgery and radiotherapy are the mainstays of treatment for STS; the role for adjuvant chemotherapy is less well-defined [2]. Less than 10% of patients present with distant metastasis at the time of initial diagnosis [3]. However, 25–50% of patients with initially localized disease may ultimately develop distant metastasis [4]. Chemotherapeutic drugs approved by the US Food and Drug Administration for the treatment of STS include anthracyclines such as doxorubicin as well as other agents such as eribulin, trabectedin, and ifosfamide, but convincing data demonstrating improved outcomes is lacking in the adjuvant setting, although studies are frequently underpowered for meaningful analysis [5, 6]. In a meta-analysis, adjuvant chemotherapy including doxorubicin was associated with increased time to local and distant recurrence; however, there was no impact on overall survival (HR, 0.89; 95% CI, 0.76–1.03; *P *= 0.12) [7]. An updated meta-analysis, including four additional trials, also found reduced overall recurrence (*P *= 0.0001) with either adjuvant doxorubicin alone, adjuvant doxorubicin-based chemotherapy, or adjuvant doxorubicin-based chemotherapy combined with ifosfamide [8]. In this updated analysis, a reduction in overall mortality in patients receiving adjuvant chemotherapy was now detected (HR, 0.77; 95% CI, 0.64–0.93; *P *= 0.01), although the impact was relatively small with an absolute risk reduction from 46 to 40%. In a pooled analysis of data from two trials not included in the updated meta-analysis, adjuvant chemotherapy was once again associated with decreased risk for relapse (HR, 0.74; 95% CI, 0.60–0.92; *P *= 0.0056), but not overall survival [9]. Interestingly, in patients that received only marginal resection of their tumor, adjuvant chemotherapy was associated with improved survival (10-year OS, 44.7% vs 27.6%; *P *= 0.048). Response rates in the neoadjuvant setting are generally 30% or less [10–13]. Little progress has been made with respect to targeted therapeutics, although the multi-tyrosine kinase inhibitor pazopanib is approved for adults with advanced soft tissue sarcoma that have received prior chemotherapy [14].

**Fig. 1:**
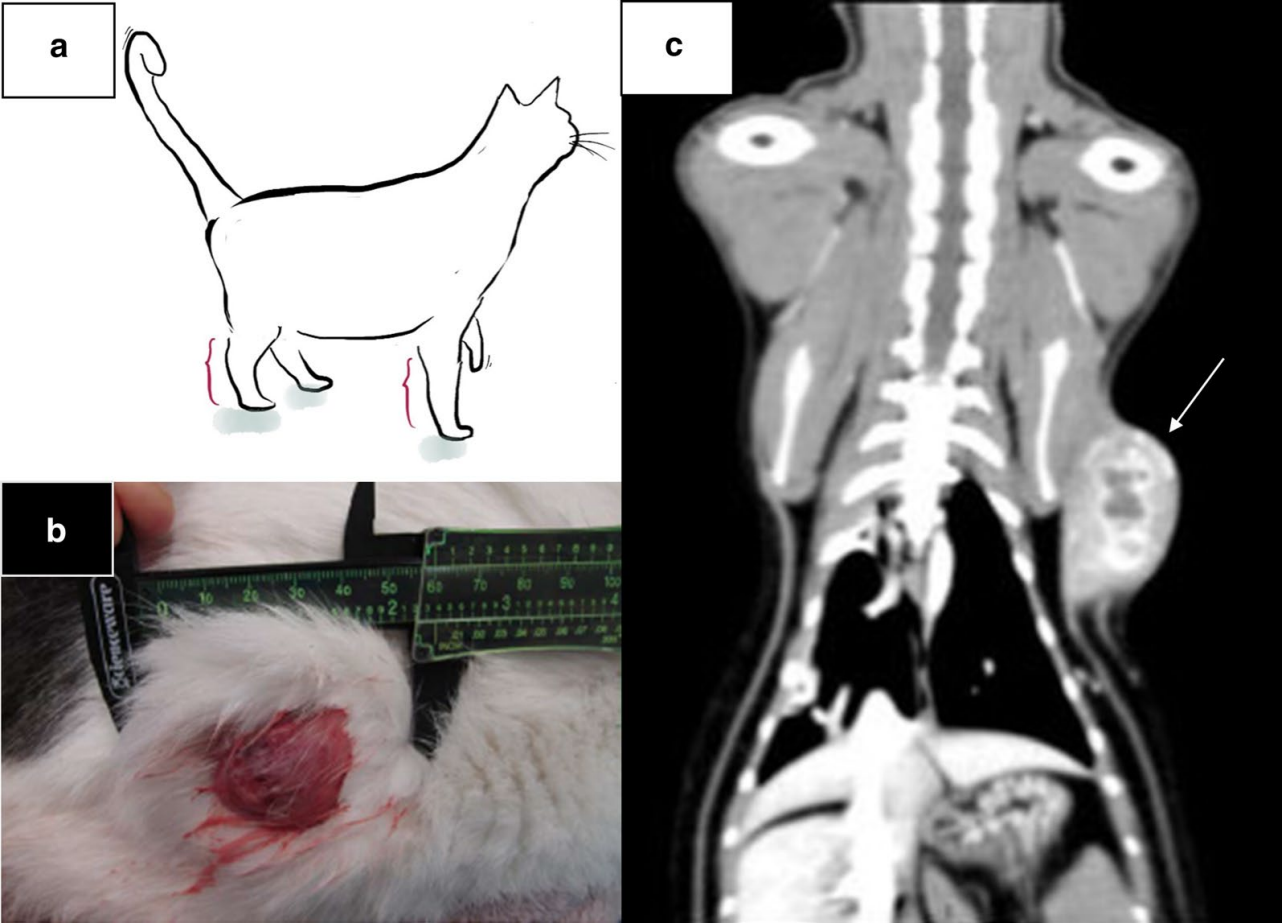
FISS are malignant, locally invasive tumors. **a** The brackets in the cat illustration depict the recommended sites for vaccine administration on the distal limbs. In the event that FISS develops, the tumor can be treated with limb amputation. **b** Photograph of FISS on the right hip of a 3 year-old male castrated Domestic Shorthair cat. **c** Coronal reconstruction of a CT scan of FISS in the subcutaneous tissues of the left thorax of a 9 year-old female spayed Domestic Shorthair cat. The arrow is pointing to the tumor

Feline injection-site sarcoma (FISS) is a type of STS that can occur in domestic cats at the site of administration of vaccines and other injections, such as antibiotics and steroids [15–17]. Like other STS, these are malignant, locally invasive tumors (Fig. 1). Metastasis to the lungs occurs in about 20% of cats [18–22]. As in people, local recurrence remains problematic in the absence of radical surgical procedures [19, 20, 22–26]. The role of chemotherapy in the management of FISS has also been investigated and remains unclear. The use of chemotherapy in macroscopic disease settings has response rates ranging from 17 to 50%, but these responses are often short-lived with a median time to progression of 84–125 days, although survival is prolonged in cats that respond to chemotherapy [27–30]. Outcomes have been variable for cats that have received chemotherapy in the adjuvant setting. Adjuvant doxorubicin was associated with prolonged survival (median, 29 months versus 5 months; *P *= 0.04) in 5 of 17 cats with macroscopic disease that had also received coarse fractionated radiotherapy [31]. In 75 cats with microscopic disease receiving either liposome-encapsulated or free doxorubicin, disease free interval was prolonged (median, 388 days vs 93 days in historical control group; *P *< 0.0001) [27]. Overall survival did not differ, but this was not a primary study endpoint due to the use of additional therapies. The combination of preoperative radiotherapy, surgical excision, and adjuvant carboplatin chemotherapy was associated with a median time to first event > 986 days (*N *= 19), but this was not statistically different from cats that received no chemotherapy (584 days, *N *= 59) or cats that received other chemotherapeutics (365 days, *N *= 14) [20]. Other studies have shown no effect of adjunctive doxorubicin or carboplatin on either local tumor control or survival time [18, 21, 32, 33]. Limitations that complicate interpretation of some of these results include small sample size, non-randomized treatment groups, retrospective evaluations, and absence of power analysis.

Predictors of chemosensitivity and chemoresistance in STS and FISS are needed in order to “target” chemotherapy to patients that benefit from these drugs. Traditional chemotherapeutics cause cytotoxicity through direct or indirect DNA interactions that result in DNA damage and cell death. Whether DNA damage from chemotherapeutics results in cell death depends upon appropriate cell-cycle checkpoint and DNA damage response (DDR) activities. Following DNA damage, kinases such as ATM (ataxia-telangiectasia mutated), ATR (ATM and Rad3-related), and DNA-PKcs (DNA-dependent protein kinase and catalytic subunit) phosphorylate H2AX, a variant of histone H2A, to form γH2AX at the site of DNA strand breaks [34, 35]. γH2AX molecules accumulate around the break and aid in the recruitment of downstream factors that initiate chromatin remodeling and break repair [36]. This accumulation is a relatively early step in the cellular response to DNA damage and detection of γH2AX can thus be used as biological indicator of DNA damage. Another important protein in the cellular response to DNA damage is p53, which is also phosphorylated by ATM, ATR, and DNA-PKcs [35]. Through transcriptional activity, activated or stabilized p53 can trigger cell cycle arrest, DNA repair and/or cell death [37, 38]. Increased activity of cellular DDR mechanisms may lead to chemoresistance, while chemotherapy has been shown to have more potent cytotoxic effects when given to patients with impaired DDR activity, at least in various epithelial cancers [39–43]. Kang et al. found that FISS tumors have variable levels of DNA damage [44], prompting us to hypothesize that tumors with higher levels of DNA damage would be more resistant to chemotherapy. To test our hypothesis, we established FISS cell lines from tumors with different levels of DNA damage and assayed for sensitivity to doxorubicin and carboplatin chemotherapeutics. We compared the chemosensitivity of the cell lines to γH2AX and *p53* expression in the original tumors.

## Methods

All animal procedures were performed according to an approved Institutional Animal Care and Use Committee (IACUC) protocol (#2011-0112).

### Acquisition of tumor specimens

Biopsy specimens were collected prospectively from client-owned cats suspected to have FISS based on clinical history, physical examination results, and diagnostic testing. Tissue collection methods were performed as described previously [44]. Sample collection and processing methods were the same for all cats. Adjacent biopsy samples from each tumor were fixed in formalin, placed into RNAlater (Sigma), or used to generate cell lines. A diagnosis of fibrosarcoma was confirmed with analysis of formalin-fixed sections stained with hematoxylin and eosin by pathologists at the Cornell University Animal Health Diagnostic Center (Ithaca, NY).

### Immunohistochemistry

Tumor samples were fixed in formalin, embedded in paraffin, and cut into 5 μm sections. Immunohistochemical staining of γH2AX was performed as previously described, using a dilution of 1:200 [44]. As reported previously, tissue sections were incubated with monoclonal mouse anti-phospho-Histone H2A.X antibody (Millipore 05–636) overnight at 4 °C, followed by a 30 min incubation with anti-mouse biotinylated secondary antibody (Invitrogen 956543B), and DAB peroxidase immunodetection (Invitrogen 002014) according to manufacturer’s instructions. The primary antibody we used was previously validated for use in cats using Western blot [45]. For quantification, three randomly selected 5 µm sections were stained from each tumor specimen. For each slide, cells with (i.e. positive) and without (i.e. negative) nuclear staining in three random non-adjacent areas were counted, and results from the 3 slides were averaged to generate a percentage of positive cells per tumor.

### p53 expression

Tissue samples in RNAlater (Sigma) were stored according to manufacturer’s instructions. Total RNA was extracted with TRIzol ™ Reagent (LifeTechnologies) per manufacturer’s protocol. Tissues (< 20 mg) were homogenized with 350 µl of TRIzol with TissueLyser (Qiagen). RNA concentration and quality were measured with NanoDrop ND-1000 instrument (Thermo Fisher Scientific). Reverse Transcriptase PCR was performed with High-Capacity cDNA Reverse Transcription Kit (Applied Biosystems) according to manufacturer’s instructions. cDNA was synthesized from 250 ng of total RNA. Real time PCR was performed with SsoAdvanced™ Universal SYBR^®^ Green Supermix (Bio-Rad) in CFX96 Touch Real-Time PCR Detection System (Bio-Rad), using thermocycler conditions of 95 °C for 30 s, followed by 40 cycles of: 95 °C for 10 s, 60 °C for 30 s. All samples were evaluated in triplicate, with gene expression reported using the ∆C_T_ method [46]. Wild type *p53* expression (Fwd primer: GCGCCTATGGTTTCCATTTA, Rev primer: GGCAAAACAGCTTGTTGAGG) was compared to *ACTB* expression (Fwd primer: CAACCGTGAGAAGATGACTCAGA, Rev primer: CCCAGAGTCCATGACAATACCA) for each replicate [47, 48].

### Generation of cell lines

Cell lines were generated using aseptic methods in a biosafety cabinet. Tissues collected were washed in sterile DPBS 1× (Corning), incubated with trypsin (Corning), then cut into approximately 2 mm pieces and plated individually onto 12-well tissue culture plates initially. Explants were monitored for cellular migration and replication. Adherent cells were subsequently passaged into progressively larger plates and eventually maintained in 10 cm tissue culture plates. The cells were maintained in standard conditions (6% CO_2_, 37 °C) in an incubator and passaged until confirmation of spontaneous immortalization and continued exponential growth. Cells were maintained in DMEM (Corning-Cellgro) with 20% FBS (Fetal bovine serum, Sigma) and 1% supplements (antibiotic–antimycotic solution, l-glutamine, MEM nonessential amino acids; Corning-Cellgro). Trypsin was used to release adherent cells.

### Chemotherapeutics

Doxorubicin (Sigma D1515) and carboplatin (Sigma C2538) were purchased in powder form. Stock solutions were prepared (doxorubicin, 2 μg/μl in sterile saline; carboplatin, 1 μg/μl in sterile water) and stored at − 20 °C until use.

### Colony forming assays

Cells were plated at variable densities (range, 4000–20,000 cells/plate, which equaled 400–2000 cells/ml; median, 8250 cells/plate) to achieve equivalent coverage by colonies in control plates at the end of the experiment (coverage range, 10–37%; median, 20%). Cells were allowed to adhere for 24 h under standard conditions and were then exposed to either low or high dose doxorubicin (0.02 and 0.03 μg/ml, respectively) or low or high dose carboplatin (2 and 4 μg/ml, respectively) in serum free cell culture medium for 24 h. Control plates were exposed to an equivalent amount of saline (for doxorubicin experiments) or water (for carboplatin experiments) in serum free medium. After 24 h of drug or vehicle exposure, drug- or vehicle-containing medium was removed, and cells were then incubated under standard conditions in standard medium. The colonies were allowed to grow until adequate colony formation was observed in control plates (range, 10–24 days; median, 11 days). Since the cell lines were established from different cats, the growth and behavior of the cells in culture differed, so variable experiment lengths allowed equivalent colony formation in control plates. At the end of the experiment, the cells were washed with 1X DPBS, fixed in methanol, and stained with 0.1% crystal violet (Sigma C3886) in 95% ethanol. Each experimental condition (i.e. control, low dose, high dose) was evaluated in triplicate per experiment. Results reflect two independent experiments for each cell line.

For colony quantification, the stained plates were scanned with an Epson V700 scanner. The images were analyzed with ImageJ [49], measuring the percentage of the plate that was covered by colonies of more than 50 cells. The chemoresistance for each low or high dose replicate is reported as relative survival compared to the mean survival of untreated controls. This automated method was used rather than manual counting in order to minimize bias.

### Xenograft model

In order to validate the ability of our cell lines to form sarcomas in vivo, subcutaneous xenografts in mice were evaluated using methods previously described (Borlle et al., BMC Veterinary Research 2018, in review). Female, heterozygous CD-1 nude mice (Charles River, Strain code 087) were crossed with male, homozygous CD-1 nude mice (Charles River, Strain code 086). Resulting, male, athymic, nude littermates were injected subcutaneously in the right flank with 5 × 10^6^ FISS cells of a given cell line suspended in 200 μl of a 1:1 solution of PBS and Matrigel (Corning) for each cell line. As a control, 200 μl of a 1:1 solution of PBS and Matrigel (Corning) without any cells was injected subcutaneously in the left flank. Tumor growth was monitored for up to 24 weeks. At the end of the monitoring period, mice were euthanized via carbon dioxide asphyxiation and necropsies were performed to collect tumor tissue. Tissues were fixed in 4% paraformaldehyde, embedded in paraffin, and stained with hematoxylin and eosin for histopathologic evaluation.

### Vimentin immunocytochemistry

Cells were harvested from plates with trypsin (Corning), spun down, resuspended in PBS, smeared, air-dried and fixed in acetone. For antigen retrieval, slides were placed in sodium citrate buffer (pH 6.0) in a pressure cooker for 2 min in a microwave oven at full power. A 3% peroxidase blocking solution (30% H_2_O_2_ in methanol) was used to reduce endogenous peroxidase activity. After peroxidase quenching, slides were blocked for 30 min at room temperature using a homemade blocking reagent (4% BSA and 0.02% Tween). Primary immunostaining (Anti-Vimentin antibody (1:100), Abcam ab8069, Cambridge, MA) was performed for 7 min at 37 °C. Secondary biotinylated antibody and detection steps were followed according to manufacturer’s instructions (VECTASTAIN^®^ Elite^®^ ABC-HRP Kit Peroxidase, Universal, PK-6200, Vector Laboratories, Inc., Burlingame, CA). The chromogenic reaction was accomplished using the Pierce™ DAB Substrate Kit (Cat#34002 Thermo Fisher Scientific, Rockford, IL). Hematoxylin was used as a counterstain.

### Statistical analysis

To evaluate for an association of γH2AX or *p53* expression in the initial tumor samples with relative survival after chemotherapy exposure in the generated cell lines, mixed model analyses were performed. Associations for each drug (i.e. doxorubicin or carboplatin) were evaluated separately. This analysis was performed using statistical software (JMP, Version < JMP Pro 13.1.0 > ; SAS Institute Inc., 2016). Cell line was a random effect within the model, and the response variable was relative survival from the colony forming assays. γH2AX and *p53* expression were evaluated as separate fixed effects within the model. If an association was detected with *P *< 0.1, additional models were evaluated incorporating other variables as fixed effects. These variables included dosage group (low vs high), initial plating density (# cells/plate), and control plate coverage (mean area [%] of control plates covered with colonies). Transformation of the response variable was performed as necessary to fit model assumptions. Significance was defined as *P *< 0.05.

## Results

### Sarcoma specimens

Results from 11 different FISS are reported in this study. Certain details on 8 of these tumors are also reported elsewhere [44]. The median age of the affected cats was 11 years (range, 3–14 years). Four cats were castrated males, and seven were spayed females. Tumor measurements were available for 10 cats, and the median tumor measurement at the longest diameter was 4.5 cm (range, 1.8–8.5 cm). Tumor location included right hip/hindlimb (*N *= 4), left hip/hindlimb (*N *= 4), left forelimb (*N *= 2), and interscapular (*N *= 1). Thoracic radiographs were performed in 7 cats, and no pulmonary metastasis was present at the time of specimen collection. One cat (sample ID, Sh4) did not have thoracic radiography performed but was confirmed to have pulmonary metastasis upon histopathologic evaluation of the lungs. Whether pulmonary metastasis was present in the other 3 cats is unknown. All tumors were diagnosed as fibrosarcomas on histopathology. Mitotic index (# of mitotic figures/10 hpf) was determined for all 11 specimens and ranged from 0 to 46 with a median of 17. Eight cats had not received any anti-cancer treatment prior to presentation and tissue sampling. The other 3 cats had received various anti-cancer therapies prior to sample collection for the current study. One of these cats was treated with surgery alone, one cat also received local chemotherapy, and the third cat received both adjuvant radiation therapy and chemotherapy. Additional treatment details are summarized in Table 1.

**Table 1:** Summary of prior therapies for the 3 cats with recurrent FISS

Cat ID	When prior therapy occurred relative to current study	Treatment details
Sh4	4 years prior	Surgical resection
Y5	1 year prior	Surgical resection with intraoperative placement of carboplatin beads^a^
E9	3 years prior	Preoperative radiation (16 × 3 Gy) Surgical resection Alternating intravenous carboplatin and doxorubicin (3 doses each)

^a^This treatment was performed at a different referral hospital and treatment details are not available

### γH2AX and *p53* expression

**Fig. 2:**
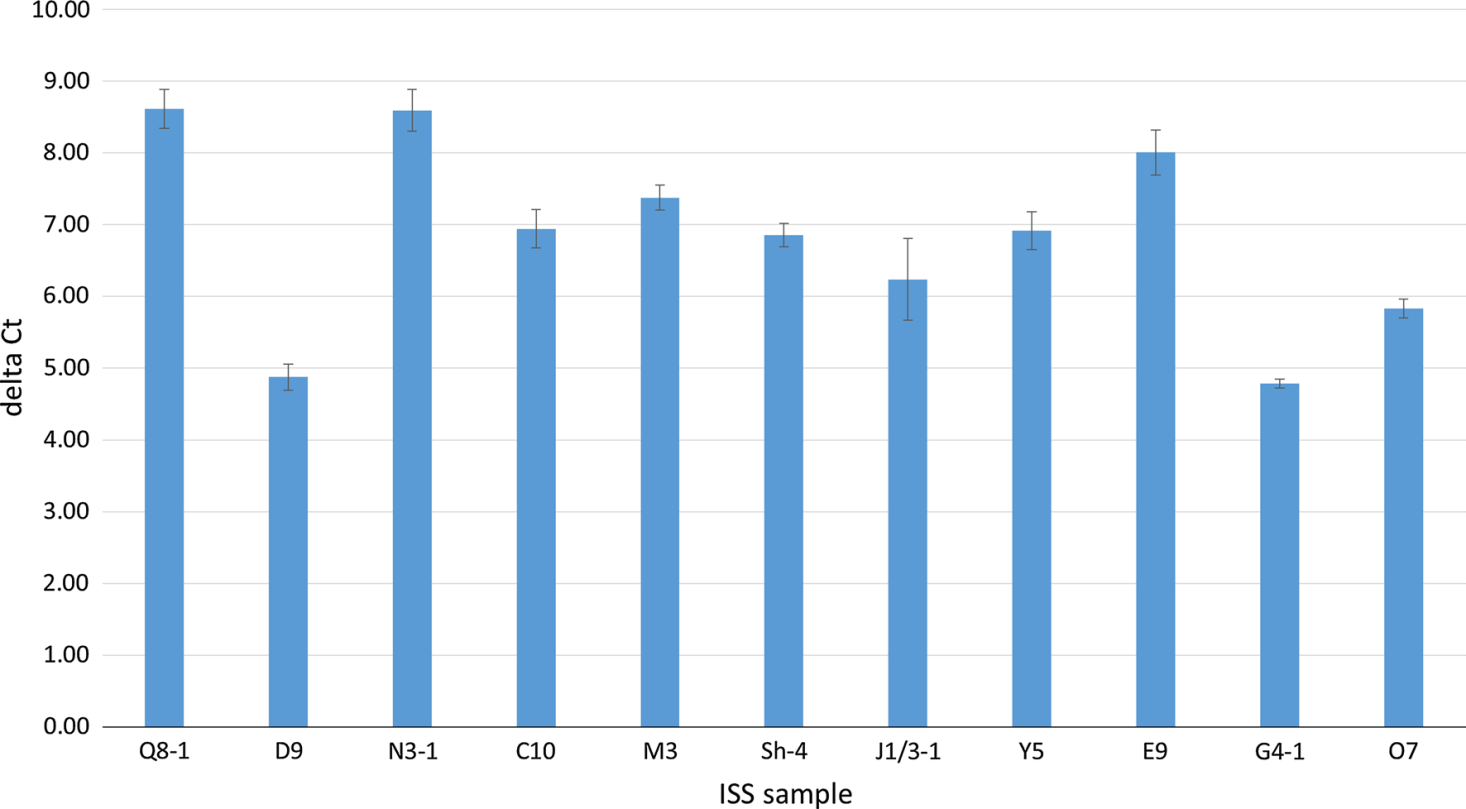
Wild type *p53* expression in feline ISS. Depicted are the mean deltaCT value ± standard deviation for each tumor sample, as compared to the housekeeping gene *ACTB*

To assess the degree of DNA damage, immunohistochemical analysis of γH2AX was performed. Nuclear immunostaining of γH2AX was noted in all specimens. The percentage of neoplastic cells staining positive for γH2AX ranged from 2.2 to 18.8% per tumor, with a mean of 13.3% (median, 14.3%). γH2AX results for 8 of the 11 samples included in the current study have also been reported elsewhere [44]. Because p53 is involved in the cellular response to DNA damage, wild type *p53* expression was also evaluated in the same tumors. Using qRT-PCR, all tumors were found to express wild type *p53* (Fig. 2). DeltaCT (wild type *p53* expression as compared to the housekeeping gene, *ACTB*) ranged from 4.79 to 8.62, with a median of 6.92.

### Associations with cell survival after carboplatin but not doxorubicin exposure

**Fig. 3:**
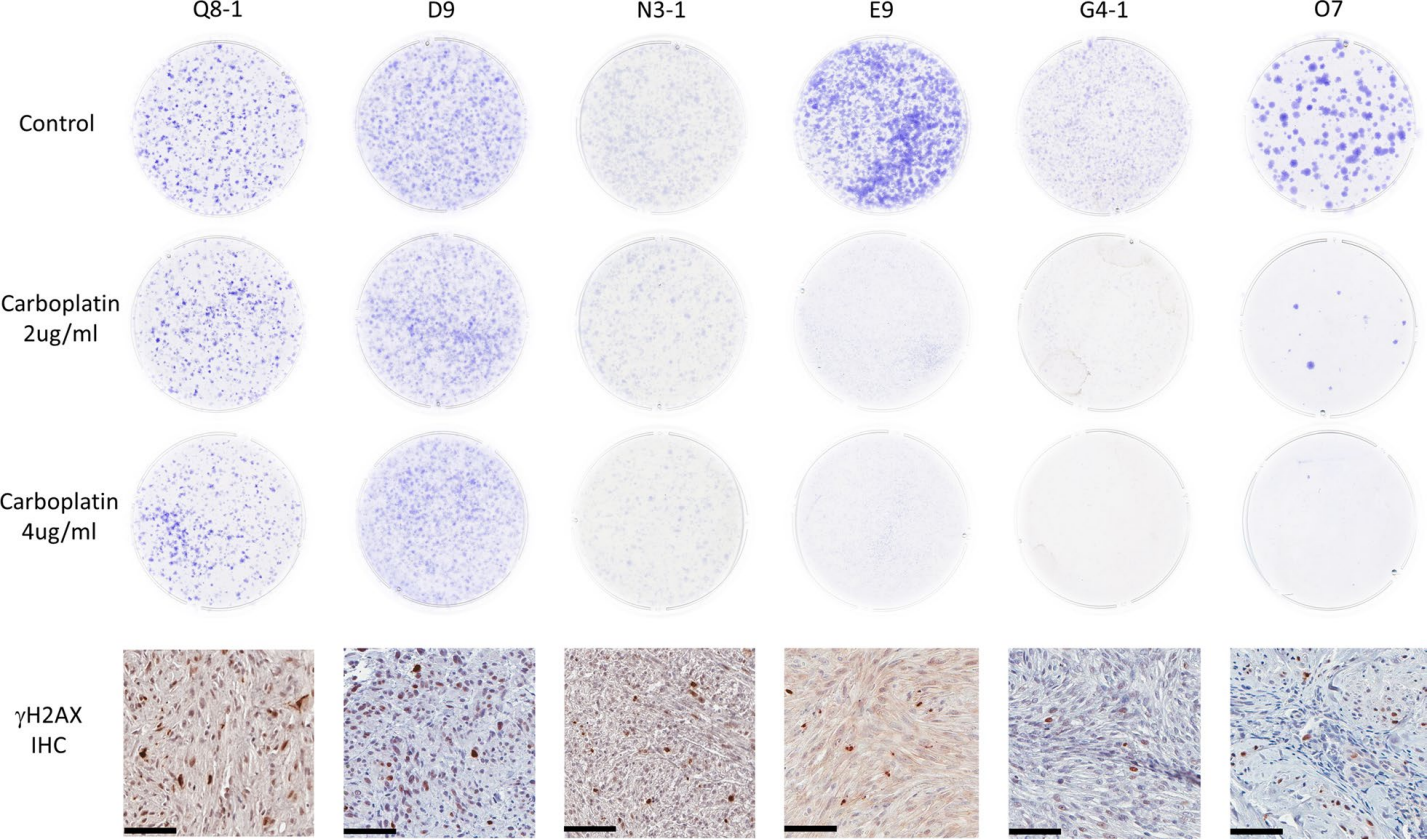
Representative colony forming assays and γH2AX expression in the corresponding tumor of origin. Shown are representative scans of colony growth for each treatment group of three carboplatin-resistant cell lines (Q8-1, D9, and N3-1) and three carboplatin sensitive cell lines (E9, G4-1, and O7). To generate images, 10 cm plates were scanned with an Epson V700 scanner. A representative image of γH2AX expression in the corresponding tumor of origin is also depicted. Scale bar = 60 µm. Digitized images were obtained with an Aperio Scanscope CS0

The ability of FISS cells to survive chemotherapy exposure and grow as colonies was used as a measure of tumor chemoresistance. Relative survival after carboplatin was determined for all 11 cell lines, and ranged from 0 to 115% (mean, 31%; median, 27%). Figure 3 shows representative results of colony forming assays from the most carboplatin-resistant cell lines (Q8-1, D9, and N3-1) and the most carboplatin-sensitive cell lines (E9, G4-1, and O7). Summary results for all cell lines are presented in Table 2. Mean relative survival after carboplatin exposure was 38% in cell lines established from tumors with γH2AX expression greater than the sample population mean of 13.3%, compared to mean relative survival of 10% in cell lines established from tumors with γH2AX expression less than 13.3%. When γH2AX and *p53* expression were incorporated as individual fixed effects within a mixed model analysis testing for associations with relative survival after carboplatin exposure, γH2AX expression was associated with relative survival (*P *= 0.0575), but *p53* expression was not (*P *= 0.1608). Additional variables that may have influenced survival were also evaluated for potential associations, including carboplatin dosage group, initial plating density, and control plate coverage. When evaluated as individual fixed effects in separate models, each of these variables had an association with relative survival after carboplatin exposure (*P *< 0.001). When all four variables of interest were included as fixed effects within the same model, each variable retained its association with relative survival (γH2AX expression, *P *= 0.0250; carboplatin dosage group, *P *< 0.0001; initial plating density, *P *< 0.0104; control plate coverage, *P *< 0.0001). Estimates and standard errors for this statistical model are provided in Table 3.

**Table 2:** Chemosensitivity of FISS cell lines to carboplatin and histologic features of original, corresponding tumor

Cell line (same as matching cat/tumor ID)	In vitro chemosensitivity		Histologic features		
	Relative survival after 2 μg/ml carboplatin (%)^a^	Relative survival after 4 μg/ml carboplatin (%)^a^	Mitotic index (#/10 hpf)	γH2AX (%)^b^	Wild type *p53* (ΔC_t_)^c^
Q8-1	92 (7)	72 (4)	19	17.6	8.62
D9	54 (13)	49 (9)	46	17.8	4.88
N3-1	57 (3)	32 (1)	6	18.2	8.59
C10	57 (12)	28 (12)	19	13.4	6.94
M3	42 (10)	19 (8)	40	14.3	7.38
Sh4^d^	40 (4)	18 (3)	17	13.9	6.86
J1/3-1	40 (9)	12 (4)	17	18.8	6.24
Y5^d^	21 (9)	13 (6)	1	2.2	6.92
E9^d^	17 (8)	4 (2)	10	9.0	8.01
G4-1	3 (1)	1 (1)	12	4.6	4.79
O7	2 (1)	0 (0)	0	16.2	5.83

^a^Mean survival results relative to matched, untreated, control cells are listed, with standard error of the mean in parentheses. Values were calculated from two independent experiments with three replicates per experiment (JMP, Version < JMP Pro 13.1.0 > ; SAS Institute Inc., 2016)

^b^γH2AX expression was determined by immunohistochemistry

^c^Wild type *p53* expression was determined by qRT-PCR

^d^Recurrent tumor. See text and Table 1

**Table 3:** Results of mixed model analysis of associations with relative survival after carboplatin exposure

Variable (fixed effect)	Estimate^a^	Standard error
γH2AX expression	3.37	1.27
Carboplatin dosage group	0.08	0.01
Initial plating density	1.29 × 10^−5^	4.95 × 10^−6^
Control plate coverage	− 0.01	0.00

^a^Note, a square root transformation of the response variable (relative survival after carboplatin exposure) was performed to meet model assumptions

Relative survival after doxorubicin was calculated for 5 cell lines (D9, G4-1, M3, Q8-1, and Sh4), and ranged from 0 to 99% (mean, 10%; median, 0.2%). Summary results for these 5 cell lines are presented in Table 4. Statistical testing revealed no association between relative survival after doxorubicin exposure and either γH2AX expression (*P *= 0.6124) or *p53* expression (*P *= 0.8645). Additional cell lines and associations were not tested. Of the cell lines where relative survival was evaluated for both carboplatin and doxorubicin, there was no obvious pattern or predictability between survival results for the two different drugs. Mean survival after doxorubicin was less than 1% for D9, Q8-1, and Sh4, whereas mean survival after carboplatin ranged from 29 to 82% for these three cell lines. Results for G4-1 and M3 are less discrepant with less than 10% difference in mean survival for the two different drugs (mean survival after carboplatin—G4-1: 2%, M3: 30%; mean survival after doxorubicin—G4-1: 10%, M3: 41%).

**Table 4:** Chemosensitivity of FISS cell lines to doxorubicin

Cell line (same as matching cat/tumor ID)	Relative survival after 0.02 μg/ml doxorubicin (%)^a^	Relative survival after 0.03 μg/ml doxorubicin (%)^a^
Q8-1	0.4 (0)	0.2
D9	0 (0)	0 (0)
M3	48 (16)	34 (13)
Sh4^b^	0 (0)	0 (0)
G4-1	15 (6)	4 (2)

^a^Mean survival results relative to matched, untreated, control cells are listed, with standard error of the mean in parentheses. Values were calculated from two independent experiments with three replicates per experiment (JMP, Version < JMP Pro 13.1.0 > ; SAS Institute Inc., 2016)

^b^Recurrent tumor. See text and Table 1

### Characterization of cell lines

**Fig. 4:**
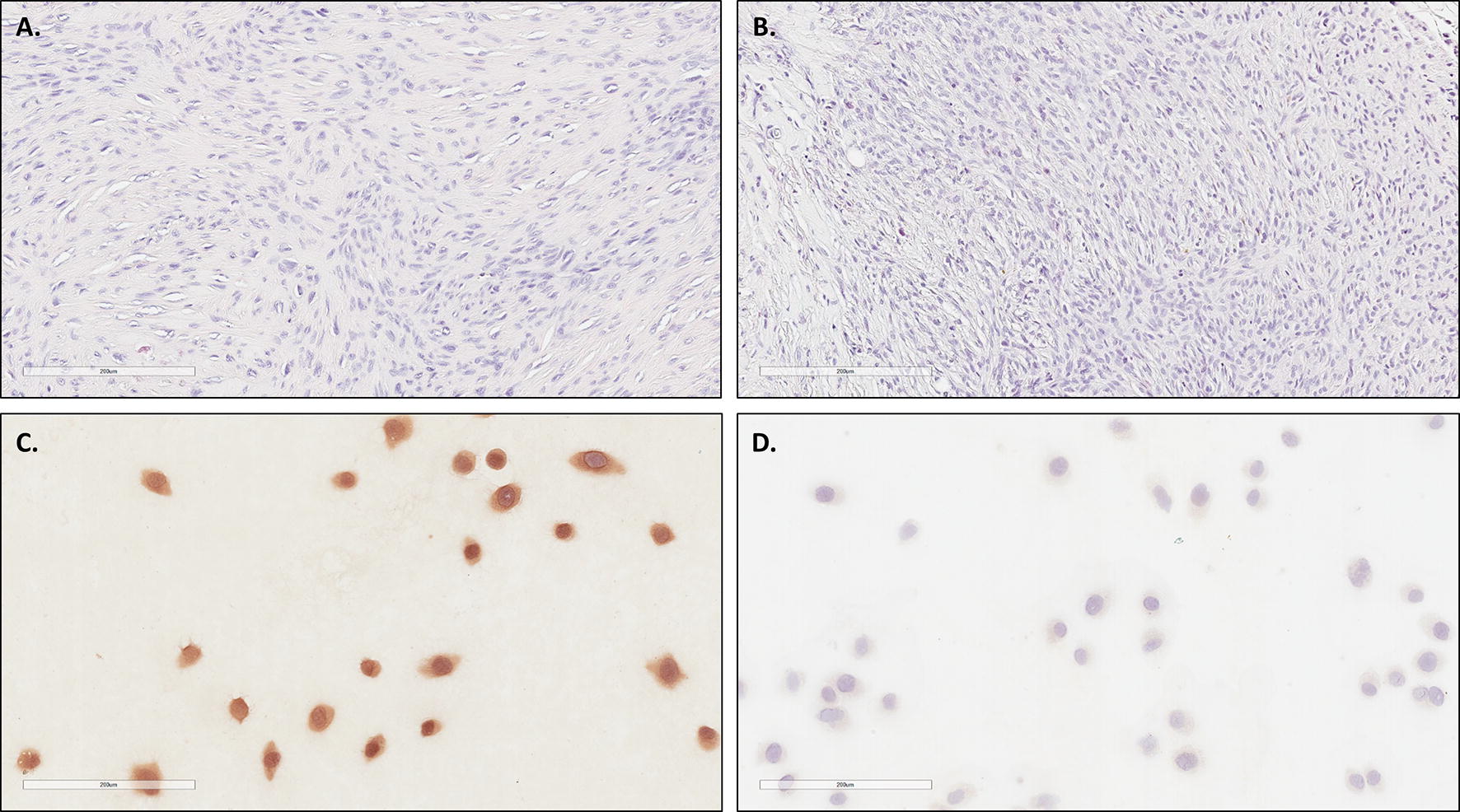
Validation of cell lines with sarcoma formation in nude mice or through detection of vimentin. Histological sections stained with hematoxylin and eosin from sarcomas that formed at the site of subcutaneous injection of E9 (**A**) and G4-1 (**B**) FISS cells. Results from C10 and J1/3-1 were similar. FISS cells from lines that were not validated by documentation of sarcoma formation in nude mice were validated via detection of vimentin expression. Results were similar for all 7 cell lines (D9, M3, N3-1, Q8-1, Sh4, O7, Y5). Depicted are representative results for D9 (**C**). **D** The corresponding negative control. Scale bars = 200 µm

The xenograft potential of 6 of the FISS cell lines was tested (C10, E9, G4-1, J1/3-1, M3, and N3-1) in male, athymic, nude mice, aged 30–122 days. After subcutaneous injection of 5 × 10^6^ FISS cells, tumor growth was monitored over 10–24 weeks. After euthanasia, tumor tissue was collected, fixed in 4% paraformaldehyde, and processed for evaluation by a veterinary pathologist (TS). Multiple mice were evaluated for each cell line. None of the mice exhibited symptoms related to the tumor during the monitoring period. A range of tumor growth was observed, depending on the cell line. Engraftment and sarcoma development occurred in 4 of the 6 cell lines tested: C10, E9, G4-1, and J1/3-1. Palpable masses grew in mice injected with M3 cells, but these regressed within 10 weeks. No tumor growth was observed in mice injected with N3-1 cells; mice were monitored for 13 weeks. Mice injected with M3 or N3-1 cells were greater than 100 days old, whereas the mice injected with the cell lines that engrafted and did not regress were 30–54 days old. Results are summarized in Table 5. Immunocytochemistry for vimentin was performed on the cell lines that did not have xenograft sarcoma confirmation (i.e. D9, M3, N3-1, O7, Q8-1, Sh4, and Y5) Vimentin expression was detected in all 7 cell lines. Representative results of sarcoma formation and vimentin expression are depicted in Fig. 4.

**Table 5:** Summary of xenograft evaluation

Cell line	No. of mice injected	No. of mice that developed masses at cell injection site	Maximum individual tumor diameter (mm)	Histological results
C10^a^	3	3	17	2 sarcomas (1 mass regressed)
E9	3	3	8	3 sarcomas
G4-1	4	4	13	3 sarcomas (1 mass regressed)
J1/3-1	5	5	15	5 sarcomas
M3	4	2	11	N/A (All masses regressed)
N3-1	2	0	N/A	N/A (No masses formed)

^a^These xenograft results are also described in Borlle et al. BMC Veterinary Research 2018, under review

## Discussion

Based on the variable level of DNA damage in FISS and the inconsistent response of these tumors to chemotherapy, we sought to understand whether the degree of DNA damage (γH2AX expression) and the response to DNA damage (*p53* expression) could be used to predict chemoresistance in FISS. In our study, cells from tumors with higher levels of γH2AX were more resistant to carboplatin, whereas there was no association with γH2AX and resistance to doxorubicin. *p53* expression was not associated with resistance or sensitivity to either drug. As none of the cats in our study had received DNA-damaging therapy within 1 year of sample collection, the γH2AX expression we detected represents endogenous DNA damage that neoplastic cells experience from replication stress, reactive oxygen species, and oncogene activation [50, 51]. When compared to tumors with lower γH2AX, tumors with increased γH2AX may have upregulation of DNA repair factors and more robust repair mechanisms allowing them to overcome the cytotoxic effects of carboplatin.

Both carboplatin and doxorubicin have been used in the treatment of FISS [18, 20, 21, 23–25, 27, 28, 31–33, 52–54]. Reports of doxorubicin use are more widespread in the literature; however, no studies have prospectively compared the two drugs. Myelosuppression, anorexia, and weight loss can occur with both carboplatin and doxorubicin, but the potential for renal damage limits doxorubicin use in some cats [55–57]. Another common platinum chemotherapeutic, cisplatin, causes fatal pulmonary edema in cats and cannot be used systemically in this species [58]. Our results suggest there may be a subset of cats with FISS that would benefit from carboplatin chemotherapy. Carboplatin is not an FDA-approved drug for STS, but it has been investigated in various clinical settings. Combination therapy with carboplatin has not demonstrated superior outcomes when compared to standard regimens for childhood STS [59–62]. Results seem more promising with improved outcomes seen with carboplatin use in patients with Ewing sarcoma or rhabdomyosarcoma with embryonal histology [63, 64]. Given promising results in certain histologic subtypes, and lack of obvious inferiority compared to standard regimens, it seems likely there is also a subset of STS patients that would benefit from carboplatin chemotherapy.

Our results were likely influenced by the drug concentrations tested, and our findings may have differed if we had evaluated markedly lower or higher drug concentrations, with uniform sensitivity or resistance, respectively, as determined by survival after treatment. One pharmacokinetic study of carboplatin in cats found that following a single intravenous bolus of 200 mg/m^2^, mean maximum plasma concentration (C_max_) was 0.023 mg/ml, and mean area under the concentration-versus-time curve (AUC) was 2.5 min*mg/ml [52]. The carboplatin concentrations used in our experiments are below this C_max_. The reported AUC is similar to a 24-h exposure of the 2 μg/ml carboplatin used in our study, suggesting this level of drug exposure could potentially be achieved in vivo. Given that we saw meaningful results with concentrations of carboplatin that can be achieved in vivo, additional testing of our hypothesis is indicated in a more clinically relevant, translational setting. In a pharmacokinetic study of doxorubicin in cats, 11 cats were given a single dose of either 25 mg/m^2^ or 1 mg/kg of doxorubicin over 10, 15 and 20 min. C_max_ ranged from 603 to 2784 ng/ml (median 1547 ng/ml), and AUC ranged from 11,436 to 70,533 min*ng/ml (median 41,519 min*ng/ml) [65]. Both of these parameters are higher than the concentrations of doxorubicin used in our experiments. However, use of higher doxorubicin concentrations in our assays is unlikely to have been informative since mean and median relative survival were already quite low.

It is not entirely unsurprising that our findings differed between the two chemotherapeutic drugs, given their disparate mechanisms of action. After activation via aquation reactions, drugs in the platinum family such as carboplatin bind tightly to DNA, forming a variety of structural adducts including intra- and interstrand crosslinks that can cause replication fork stalling [66]. These stalled replication forks can lead to double strand breaks and cell death. It is the intrastrand adducts that are primarily responsible for cytotoxicity; as such, it is the degree of successful nucleotide excision repair (NER) that is responsible for cell survival [67]. In contrast, the primary mechanism of cytotoxicity and antitumor activity of doxorubicin, an anthracycline antibiotic, is to inhibit the action of topoisomerase II, an enzyme that is essential for DNA synthesis. Topoisomerase II inhibitors stabilize DNA cleavage complexes which results in persistent double strand breaks that can cause cell death [68]. Additionally, redox cycling of doxorubicin generates reactive oxygen species that can cause further damage [69]. Due to the complexity of lesions that can result, DNA repair after damage from doxorubicin can involve a variety of repair processes, including NER and non-homologous end joining [69, 70]. Alterations in NER are well-recognized as a mechanism of carboplatin resistance [67], whereas doxorubicin resistance is most often associated with increases in P-glycoprotein, a membranous pump that transports doxorubicin out of cells [71, 72]. Variations in topoisomerase IIα can also contribute to doxorubicin resistance [73]. Unlike our findings with the anthracycline doxorubicin in FISS, the presence of γH2AX foci in breast cancer cells was associated with resistance to a different anthracycline chemotherapeutic, epirubicin [40]. Although it is possible that alterations in DDR pathways are not involved in resistance to doxorubicin in the FISS cells we investigated, we analyzed a limited number of cell lines, and these particular cell lines were rather sensitive to the doxorubicin concentrations used, limiting the overall power of this aspect of our analysis.

In contrast to the association we detected between γH2AX expression and carboplatin resistance, we found no association with *p53* expression and FISS cell survival after either carboplatin or doxorubicin exposure, although we were able to detect wild type *p53* in all tumors. Under standard conditions, most cells express low levels of p53, with p53 normally being a very short lived protein at a barely detectable level [38, 74]. When DNA damage or other cellular stresses occur, the amount of p53 protein increases rapidly via stabilization rather than increased synthesis. The evolutionarily conserved DNA-binding domain of *p53* is the most frequent site of somatic mutations in various human cancers and is typically associated with aggressive phenotypes [38]. *p53* mutations have been detected in up to 50% of human STS tumors, with mutations occurring more frequently in metastatic sarcomas and high-grade lesions [75–77]. Interestingly, feline neoplasms do not seem to harbor *p53* mutations with the same frequency as that observed in man [78–81]. Despite this, aberrant *p53* expression has been reported in FISS. Somatic allelic deletion as evidenced by loss of heterozygosity at *p53* was detected in 60% of primary FISS, which was associated with rapid tumor recurrence and reduced overall survival [82]. In a separate study, cytoplasmic expression of p53 was associated with shorter time to tumor recurrence compared to those cats with tumors exhibiting nuclear p53 staining [83]. We did not evaluate whether any tumors in our study had *p53* mutations, and this may have been why we did not detect any associations with cell survival. However, wild type *p53* does seem to influence chemosensitivity under certain circumstances. Cytotoxicity of a variety of chemotherapeutics, including doxorubicin, relies on activation of wild type p53 following DNA damage, and subsequent engagement of target genes such as BAX and PUMA [84, 85]. In STS cells harboring *p53* mutations, reintroduction of wild type *p53* enhanced chemosensitivity to doxorubicin through inhibition of MDR-1 P-glycoprotein expression [86]. A dose response relationship has also been demonstrated for wild type p53 and response to a UV mimetic in isogenic murine teratocarcinoma cells [87]. Promotion of the apoptotic activity of p53 may be of therapeutic benefit in solid tumors. For example, methyl-β-cyclodextrin, which removes plasma membrane cholesterol, has been reported to sensitize breast and liver cancer cells to doxorubicin by enhancing p53 protein level and its nuclear localization, leading to increased cell membrane expression of FasR and activation of the extrinsic apoptotic pathway [88].

After injection of FISS cells into nude mice, some of the cell lines in our study failed to form tumors or the tumors grew poorly. This may have been a consequence of the residual mature B cells, dendritic cells, macrophages and natural killer cells that were present and functioning in the athymic mice. Inconsistent engraftment is not uncommon with this particular mouse strain [89, 90]. Evaluation of xenografts using immunodeficient mice that are more deficient in humoral and innate immunity, for example the NOD SCID strains, should be considered for future experiments to confirm sarcomagenesis for other cell lines that grew insufficiently in the CD-1, athymic, nude mice we evaluated [91, 92].

Further validation of the association we detected between γH2AX expression and carboplatin resistance is warranted with additional in vivo studies using animal models, such as evaluating the response of our cell lines to carboplatin in a xenograft model, or through evaluation of γH2AX expression and clinical response to carboplatin in cats with spontaneous tumors. As γH2AX only represents one element of the complex DNA damage response pathway, future research evaluating other downstream proteins such as DNA repair factors 53BP1 or BRCA1 [93] may allow development of a more fine-tuned algorithm predicting chemoresistance or chemosensitivity in STS.

## Conclusions

In summary, we have demonstrated that DNA damage in FISS tumors is associated with resistance to carboplatin in FISS cell lines established from those tumors. Low γH2AX expression may therefore serve as a biological indicator of carboplatin chemosensitivity in STS. In vivo studies in animal models are indicated to further validate our findings.

## Abbreviations

ATM: ataxia-telangiectasia mutated
ATR: ATM and Rad3-related
AUC: area under the concentration-versus-time curve
C_max_: maximum plasma concentration
DDR: DNA damage response
DNA-PKcs: DNA-dependent protein kinase and catalytic subunit
FISS: feline injection-site sarcoma
IACUC: Institutional Animal Care and Use Committee
NER: nucleotide excision repair
STS: soft tissue sarcoma
